# Conference Report: Workshop on Water: its Measurement and Control in Vacuum Gaithersburg, MD May 23–25, 1994

**DOI:** 10.6028/jres.100.009

**Published:** 1995

**Authors:** Stuart A. Tison, J. Patrick Looney

**Affiliations:** National Institute of Standards and Technology, Gaithersburg, MD 20899-0001

## 1. Introduction

A workshop on water measurement and control in vacuum environments was held at NIST on May 23–25, 1994. The objectives were to determine the present state of water vapor measurement and control technology in vacuum applications, to explore ways of industrial implementation, and to identify unsolved problems. The scope of the workshop included the assessment of the current understanding of H_2_O interaction with technical vacuum surfaces, the available techniques for modeling water outgassing from vacuum surfaces, cleaning techniques and surface treatments to minimize adsorbed water, water vapor measurement techniques in vacuum, and the identification of further research which would contribute to a better understanding of water in vacuum environments. The workshop was cosponsored by the NIST Advanced Technology Program, the NIST Thermodynamics Division, and the Vacuum Technology Division of the American Vacuum Society. The audience included participants from academic, government, and industrial institutions representing a number of different disciplines and industries including the space, semiconductor, and electronic components industries as well as a number of manufacturers of vacuum equipment and instrumentation.

The workshop was composed of 35 oral presentations which occurred over a two and one half day period, and one half day of small group discussions on water-surface physics, surface modification and cleaning techniques of vacuum systems, and water vapor measurement instrumentation. Over 70 attendees were present for the workshop. The workshop presentations are listed in order of presentation in Appendix A.

The measurement of contaminant gases in vacuum environments is important for the aerospace, semiconductor, and electronics industries, among others. To set the stage for further presentations and discussions, four introductory presentations were made to identify the criticality of water vapor measurement and control in closed electrical and electronic devices, semiconductor processes and in space applications.

The problem of water vapor as a contaminant in vacuum has been known for quite some period of time [1]. Lieszkovszky pointed out that Irving Langmuir was one of the first to discover the deleterious effects of contaminant water. Langmuir found that the lifetime of a tungsten filament was reduced by the presence of water vapor which reacted with the tungsten through an oxidation cycle. He later discovered that the life of a light bulb could be increased by the addition of halogens to counteract moisture and minimize evaporation related blackening. Water has subsequently been found to be a major contaminant in sealed electronic devices, space optical systems, and semiconductor processing tools. Although the presence of water vapor in vacuum has been known for quite some time, the ability to quantify or measure the water vapor has not been well developed.

Water may be introduced into sealed electronic components with the sealing gas, released from the package material or penetrate through leaks in the enclosure. Lowry stated that water vapor, when adsorbed onto the metallic structure, can cause surface electrical leakage and chemical and galvanic reaction, which may lead to premature failure of the device [2]. He went on to explain that although increased use of plastic encapsulated microcircuits has decreased this type of failure rate, the number of water related failures are still significant in gas sealed enclosures.

Water vapor can be introduced into vacuum systems from process gases, can desorb from interior surfaces, be generated via chemical reaction or permeate through polymeric seals. Shapiro stated that the presence or residual moisture in semiconductor processing is critical for a number of processes and even 1 part per billion concentrations have been demonstrated to adversely effect 64 Mb Dynamic Random Access Memory (DRAM) production [3]. Water vapor may oxidize the surfaces of unprotected films or induce particle formation via reaction with process gases and in turn cause localized defects when deposited on semiconductor devices. Water has been identified as a major contaminant in a number of processes which include physical vapor deposition (PVD) [3], plasma etching of metal films [4], and chemical vapor deposition [5]. Consequently, it is estimated that the effect of residual water vapor upon semiconductor processing results in lower device yield rates and reliability failures, costing the semiconductor industry billions of dollars annually [6].

Uly discussed spaced-based sensors which can be affected by adsorbed water vapor which desorbs in space and adsorbs on optical or electronic systems. This absorbed water can cause optical adsorption and corrosion of electronic components. Many earth observing spacecraft detect infrared radiation over the 2 μm to 30 μm region where released water vapor from the craft surfaces produce emissions from the radiative decay of vibrationally excited states [7]. In addition, the residual upper atmosphere impacts spacecraft windward surfaces. The kinetic energy in these collisions, 3 eV to 10 eV, is sufficient to desorb molecules from surfaces, collisionally excite vibrational modes of molecules, induce chemiluminescent reactions. Although the presence of water vapor is known to affect the performance of space sensors, quantification of the amount and sources of water contamination are not well understood. A future experiment, the MSX spacecraft mission [8], will include a suite of sensors to accurately measure the local environment surrounding the spacecraft as a function of time in orbit. The experiment is expected to determine the necessary time in orbit required for proper water degassing before optical sensors are activated and will also assess the effectiveness of current ground contamination control procedures.

Albeit water contamination has numerous unique deleterious effects depending upon the process or application, the available instrumentation for its measurement and the physics of its interaction with surfaces is common to all. The workshop was broadly organized according to the following topics: water outgassing- experiments and modeling, surface modification and cleaning techniques, water vapor measurement using traditional techniques, and optical spectroscopic techniques for water vapor measurement.

## 2. Modeling of Water Outgassing

Two of the pioneers in outgassing studies, P. Redhead and B. Dayton, presented overviews of the current progress in modeling of water vapor outgassing. The types of models traditionally used can be classified as surface desorption limited and diffusion limited. In the surface desorption limited model all the water is assumed to be adsorbed on the surface and none diffused into the bulk material. The diffusion limited model conversely assumes all absorbed water exists in the bulk and diffuses to the surface where it desorbs. There was considerable debate as to the appropriateness of these models, but general agreement that both effects should be accounted for in a complete model. The surface limited model first used by Venema [9], gives rise to the following equation:

$$\ln\frac{p_{o}}{p}-\frac{N_{m}}{KV}\int_{p_{o}}^{p}p^{-1}(\frac{df}{dp})dp=\frac{t}{\tau_{p}}$$ (1)

where *K* is the number of molecules in a Pa liter, *V* the system volume in liters, *τ_p_* equal to the system volume divided by the system pumping speed with units of seconds, *p_o_* the pressure at beginning of pump down, *f* the adsorption isotherm, *t* the elapsed time in seconds and *N_m_* the total number of molecules adsorbed. Any suitable isotherm can be substituted into Eq. (1) to yield the pressure-time relationship. According to Redhead, the model that best fits the experimental data [10] for multiple water layers near ambient conditions is Henry’s law. However, for the two most inner layers a more complex isotherm, the full Tempkin isotherm, better represents the data. Additionally, water sorption at grain boundaries could be modeled as an adsorbed molecule having a long residence time in a surface site. The effective activation energy of desorption can be related to the diffusion time and employed in a Tempkin isotherm. Redhead also mentioned that Malev [11] had implemented a full outgassing model with bulk diffusion and surface desorption, although the conclusions from such a model were not general in nature and yielded little insight into the physics of outgassing.

In addition to water outgassing from interior vacuum surfaces, water in the vapor phase, introduced when the system is vented, is a major contributer to contaminant water. Liu presented data indicating that during rapid pumpdown adiabatic cooling of the gas can cause water vapor condensation producing a stable aerosol. The aerosol, when adsorbed on a device in a semiconductor tool, may cause local defects. Additional water vapor is often introduced into the vacuum system with the process gas. Because of this, modeling of water vapor in gas distribution systems is of importance. A model presented by McAndrew [12] predicts water vapor transport in a gas distribution system by assuming a particular isotherm for water adsorption.

## 3. Water Outgassing Measurements

Outgassing measurements from vacuum materials are numerous and of varying quality. While many measurements have been performed, a fundamental understanding of outgassing has not been developed. Some recent measurements have attempted to correlate outgassing rates with vacuum surface characteristics. Dylla stated that the outgassing rate from metals will be highly dependent upon the surface characteristics including the oxide layer thickness and composition, and the surface roughness. The water outgassing rate and total quantity of outgassed water as a function of pumping time depends on exposure conditions and the physical and chemical state of the passivation layer. Unfortunately these attributes are rarely quantified in outgassing studies. Measurements by Li and Dylla [13], Ishimaru et al. [14], and Chen et al. [15] have quantified the relationship between water exposure and outgassing for a number of surfaces. A recent study by Dylla et al. [16] showed little correlation between oxide thickness and surface roughness with total outgassing. However, outgassing measurements with a fixed surface roughness and varying oxide layer thicknesses show more of a correlation [17]. In all cases the total quantity of outgassed water from surfaces is more than can be accommodated on the surface of the metal which implies that the oxide layer is a large source of water from metal surfaces.

Modeling of the complex diffusion process was presented by Weiss which implemented a heuristic classical statistical mechanics model. The parameters of the model such as binding energies are calculated from previous outgassing studies to predict outgassing rates for the Laser Interferometer Gravitational Wave Observatory (LIGO).

Akbulut discussed measurements of water adsorption and desorption on oxidized tungsten, W(100). His data show an increasing binding energy of water on the surface as the coverage decreases. Additional data presented using low energy ion scattering (LEIS) indicate that a small percentage of adsorbed water dissociates when impacted by low energy ^16^O^+^ ions according to the following reaction of Eq. (2). Implications of these types of reactions are of fundamental interest in electrochemistry and interface science.

$$H_{2}^{18}O{+}^{16}O\to^{18}\text{OH}(\text{ad}){+}^{16}\text{OH}(\text{ad})$$ (2)

Although there are volumes of outgassing data from vacuum materials, fundamental measurements of the adsorption kinetics of water on stainless steel, for example, have not been accomplished. Dylla pointed out that without detailed adsorption kinetic information it is not possible to distinguish the effects of different source term assumptions in current models: water desorbing from a rough surface, from pore surfaces connected to the surface, or from diffusion from the oxide layer.

An additional source of water vapor in vacuum systems is from water diffusion through polymeric seals. Numerous data exist for diffusion of atmospheric gases such as nitrogen, oxygen, and carbon dioxide through polymers, but less is known about water permeation. Shadman presented data [18] obtained with an atmospheric pressure ionization mass spectrometer (APIMS) to measure the permeabilities of water vapor in vacuum polymers. Data presented at the workshop included the water vapor permeability temperature dependence of many polymers used in vacuum applications.

## 4. Surface Modification and Cleaning Techniques

In many instances it is necessary to minimize or quickly eliminate the adsorbed water when a vacuum system is exposed to ambient moisture. Either the material surface can be modified to minimize adsorption or a technique employed to quickly desorb the water vapor before or during pumpdown. Ishimaru discussed two techniques for minimizing or removing adsorbed water. The first uses a modification technique where the surfaces are highly polished “mirror finished” [19] to limit the amount of adsorbed water. The second uses a chemical treatment based upon the use of 2,2-dichloropropane, (CH_3_)_3_SiCl, to remove water via chemical reaction [20]. Another method for water removal presented by Li is glow discharge cleaning (GDC) which is frequently used in accelerator and fusion device vacuum systems to remove carbon and oxygen based gaseous impurities from chamber walls. He shows a ten-fold reduction in pumpdown time using a helium glow discharge cleaning technique [21].

Baragiola presented data on the ultraviolet, Lyman-α (1216 Å) photodesorption of ice between 35 K and 100 K. He discovered that the photodesorption yield was undetectable at the onset of irradiation and increased with irradiation dose up to a saturation value (0.004 molecules per photon was a typical value).

Although many cleaning techniques exist, their use in semiconductor applications has been limited by excessive time required, chemical incompatibilities or marginal gain achieved. More prevalent is the use of “mirror finishing” to reduce particle generation and gas adsorption. While mirror finishing has been proven to lessen particle generation, its ability to reduce water adsorption, or decrease the time required for system pumpdown is not universally acknowledged.

## 5. Water Vapor Measurement

The in situ measurement of water vapor is traditionally accomplished using vacuum instrumentation such as hot-filament ionization gages, quadrupole mass spectrometers, cold cathode gages, and more recently spinning rotor gages. While all of these instruments are capable of measuring water vapor, only the mass spectrometer is capable of measuring the partial pressure of water vapor which is of the most importance for monitoring its presence as a contaminant gas. Tilford presented data showing the many problems associated with using a quadrupole mass spectrometer to measure the partial pressure of water vapor. Some of the deleterious effects include sensitivity variations with total pressure, instabilities with time, pressure nonlinearities, variable response times, and gases generated via hot-filament interactions [22]. Although these instruments have many limitations for monitoring contaminant water vapor, their use is predominant in the semiconductor industry for monitoring contaminant gases in semiconductor tools. Total pressure gages such as hot-filament ionization gages and cold cathode gages have greater stability in water than many quadrupole mass spectrometers [23], although they still exhibit response time changes, and gas generation effects.

Another method for monitoring the partial pressure of water vapor uses quartz crystal microbalances (QCMs). These devices, commonly used in the space environment for accurately measuring molecular contamination, sense the accumulation of water upon their surface by monitoring the resonant frequency of a quartz crystal placed in the vacuum environment. Glassford gave an overview of the use of QCMs to measure outgassing from space materials [24]. Wallace discussed how QCMs can be used to measure the partial pressure of water vapor in process gases. Although QCMs may be valuable tools in many applications, they are generally limited by their sensitivity and their non-specificity to condensable gases.

The use of tritium-labeled water to study water transport phenomena was described by Dobrozemsky. In this technique the sample surface is exposed to a known concentration of labeled water which is latter desorbed in another vessel where the tritium decay rate is measured and correlated to a surface coverage of the material under study. Dobrozemsky estimates that fractions of a monolayer coverage could be measured with this technique [25].

While monitoring water vapor partial pressure in the process chamber is of the most importance, independent monitoring of the process gas is also necessary to prevent introduction of water contamination. Ketkar demonstrated how atmospheric pressure ionization mass spectrometry (APIMS) has been used to monitor the partial pressure of water vapor in nitrogen with concentrations as low as 60 parts per trillion [26]. Although the APIMS technique is very useful, its specificity is limited by the chemical reactions involved which make the measurement of process gases contaminated with multiple active gases difficult.

All of the previously discussed instruments require calibration for quantitative measurement. Because of water’s complicated adsorption and desorption, generation of known water vapor pressures is usually done dynamically by passing a known water flow through an orifice of known conductance. Hinkle reviewed techniques for generating known flows of water vapor using pressure based flow meters, thermal mass flow meters, saturated carrier gas techniques and liquid delivery with subsequent vaporization. Tison described a system developed at NIST for generating known water vapor pressures over a range of 10^−5^ Pa to 10^−2^ Pa, based upon a Knudsen-effusion source and a calculated conductance.

Even though many instruments are commercially available for measuring the partial pressure of water vapor, their usefulness is limited by their sensitivity, stability with time, specificity, and in some cases response time.

## 6. Optical Water Vapor Measurement Techniques

The measurement of water using traditional vacuum instrumentation, such as hot filament ionization gages or quadrupole mass spectrometers, has limitations which were previously discussed. Recently, there has been a renewed interest in the development of optical or spectroscopic techniques for the measurement of water vapor. Spectroscopic techniques have advantages which include a response proportional to the local gas density, species specific detection, and measurement times of seconds. Different techniques offer different levels of sensitivity of which several were discussed at the workshop. Three of the talks centered around the use of various absorption spectroscopies for water vapor measurement.

Hovde discussed the use of tunable diode laser absorption spectroscopy [27] for monitoring water vapor both in vacuum and in atmospheric pressure environments. In these measurements, the fraction of light absorbed by a gas sample provides a measure of the average number density in the optical path. With tunable diode lasers which operate in the near infrared, in particular the region between 1200 and 1700 nm, detection sensitivities on the order of 10^−7^ Pa to 10^−5^ Pa for a 10 m absorption path length were stated. Atkinson discussed the use of intracavity laser absorption spectroscopy (ICLAS) to monitor water in vacuum. The ICLAS technique places a cell containing water into a multimode laser cavity. The laser’s broadband emission spectrum exhibits dips, or losses, in intensity due to water vapor absorption in the laser cavity. Sensitivities for the ICLAS technique were stated by Atkinson to be below 1 ppb, possibly below 100 ppt.

A novel approach to the measurement of absorption was given by Lehmann, who discussed the use of optical “ring down” cavities [28] for water measurement. The ring down cavity uses recently developed high reflectivity dielectric mirrors (R>99.999 %) to form a high quality (or finesse) cavity. A laser pulse is used to excite the cavity, and the excited modes slowly decay with time (i.e., “ring-down”). The decay time of the light intensity in the cavity (or the ring-down time) is a direct measure of the losses in the cavity, including the absorption. Lehmann discussed the application of this technique to water vapor in the near infrared, and illustrated the high sensitivity of this technique using his work on the very weakly absorbing overtone bands of HCN as an example.

Looney discussed the use of resonance enhanced multiphoton ionization (REMPI) [29] to measure water vapor in vacuum. In this technique, a tunable UV laser is used to selectively ionize water molecules which are detected using a time-of-flight mass filter and a suitable ion detector. Looney demonstrated detection limits on the order of 10^−8^ Pa using this technique.

The highly hygroscopic nature of polyimide films generally has a deleterious effect on the adhesion properties of vacuum deposited adlayers. The season during which the film was cast, the length of time in storage and conditions during shipping all lead to variability in the water content in polyimide films. Sweitzer discussed the measurement of the water content in polyimide films using Fourier transform infrared (FTIR) spectroscopy for improved process control in the deposition of copper films onto polyimide films for flexible printed circuitry applications.

## 7. Small Group Discussions

The workshop concluded with a half day of small group discussions. These reiterated presented material, summarized the present state of water measurement and control, and identified key areas for future investigation. Three small groups were created to focus on the following areas: water outgassing models, cleaning and surface modification techniques to minimize water adsorption, and water vapor measurement instrumentation. The discussions from the small groups are summarized below.

### 7.1. Water Outgassing Models

Since most of the water outgassing models are empirical in nature, their applicability is limited to surfaces which are very much like those for which the data were obtained. It was recognized that measurement of the desorption isotherms on mechanical surfaces is necessary to make the models more robust. These determinations would also give insight into the quantity of absorbed water on the surface and that which is adsorbed into the surface oxide layer.

### 7.2. Surface Modification and Cleaning Techniques

The ability of surface modification, such as “mirror finishing,” to reduce the amount of adsorbed water vapor or to reduce the amount of time required to achieve a target pressure, is not well established. Some improvement has been shown on aluminum surfaces but the cost may not merit implementation due to marginal decreases in pumping time. Glow discharge cleaning with helium is a promising technique in applications where the presence of the plasma is compatible with the system. Other cleaning techniques using solvents, and surfactants seemed to have little effect upon water outgassing amounts. Chemical reaction processes such as those based on 2,2-dichloro-propane may be acceptable in some applications but were not well received due to the possible corrosives generated.

### 7.3. Water Measurement Instrumentation

Traditional methods for measuring water using residual gas analyzers (RGAs) have many limitations. The water sensitivity of RGAs may be affected by the presence of other active gases, the total chamber pressure, previous exposure to active gases, and instabilities with time. Additionally, the hot-filaments convert a large percentage of the water vapor into other gases such as hydrogen and carbon monoxide. However, RGAs achieve high sensitivity and are flexible in operation. QCMs are inert and can be used to measure water vapor, but are not species specific, and have limited sensitivity. Optical spectroscopic techniques, including infrared absorption, resonance enhanced multiphoton ionization, and ring down techniques, have good sensitivity and selectivity and do not employ a hot cathode. However, these techniques are generally specific to one species, are still in the development stage, and tend to be expensive.

## 9. Appendix A. Workshop Presentations

1. Trace Moisture and Its Measurement in Lighting Products—Closed Capsules, L. Lieszkovszky, General Electric
2. Water in Electronic Enclosures, R. Lowry, Harris Semiconductor
3. Effects of Water on Semiconductor Processing, A. Shapiro, Sematech/IBM
4. The Effects of Water Vapor on Space Sensor Operations, O. Uy, Johns Hopkins University Applied Physics Laboratory
5. Measurement and Modelling of the Water Outgassing from Metal Surfaces, H. Dylla, CEBAF
6. Fundamentals of Low-Temperature and High-Temperature Water Adsorption on Metal Surfaces, E. Stuve, University of Washington
7. A Possible Mechanism for the Formation of Water Molecules in Stainless Steel Vacuum Systems, T. Stack, Process Physics Incorporated
8. Atmospheric Pressure Ionization Mass Spectrometry Calibration and Measurement of Sub PPB Levels of Water in Bulk Gases, S. Ketkar, Air Products and Chemical Company
9. APIMS Measurement of Water Permeation in Polymers, F. Shadman, University of Arizona
10. The Outgassing Rate of Preconditioned Vacuum Systems after Short Exposure to the Atmosphere, Part I: Outgassing Rate Measurements on Viton-A and Copper, B. Dayton, Consulting Services
11. Condensation and Residue Particle Formation During Vacuum Pump Down, B. Liu, University of Minnesota
12. A Novel Method for the Removal of Adsorbed Water from Vacuum Systems, W. Weed, Sandia National Laboratory
13. Reduction of Outgassing Rate by Glow Discharge Cleaning, M. Li, College of William and Mary
14. Ultraviolet Photodesorption from Water Ice, R. A. Baragiola, University of Virginia
15. Modelling the Pump-down of Systems with Adsorbed Water, P. Redhead, National Research Council (Canada)
16. Water in Vacuum/Cleaning/Fast Pump-down Process, H. Ishimaru, KEK National Laboratory for High Energy Physics
17. Unique Surface Treatment of 304 Stainless Steel Vacuum Chamber Reduces Pump Down Time, J. Pernicka, Pernicka Corporation
18. Tritium Tracer Technique as a Tool for Atmospheric Water Adsorption Studies — Methodical Issue and Selected Results, R. Dobrozemsky, Austrian Research Center
19. High Sensitivity Water Detection: Intracavity Laser Spectroscopy, G. Atkinson, University of Arizona
20. Ring-Down Spectroscopy as a Means for the Detection of Trace Species, K. Lehmann, Princeton University
21. High Sensitivity Measurement of Water Vapor by Tunable Diode Laser Absorption Spectroscopy, C. Hovde, Southwest Sciences Incorporated
22. Determination of Water Content in Polyimide Film by IR, B. Sweitzer, Sheldahl Incorporated
23. Resonant Enhanced Multi-Photon Ionization of Water, P. Looney, NIST
24. The Measurement of Water Outgassing Rates Using the QCM Collection (ASTM E1559) Method, P. Glassford, Lockheed Missiles and Space Corporation
25. Use of QCMs to Measure Low Partial Pressures of Water Vapor in Process Gases, S. A. Wallace, Process Physics Incorporated
26. The Effects of Water Vapor on Vacuum Instruments, C. Tilford, NIST
27. Water Outgassing and Model for the LIGO Beam Tube, R. Weiss, Massachusetts Institute of Technology
28. Water-Vapor Primary Vacuum Standard, S. Tison, NIST
29. Properties of Vacuum Deposited Water Ice Fims, R. Baragiola, University of Virginia
30. Adsorption of Water on Oxidized Tungsten and Suppression of Electron Induced Oxygen Ion Emmision by Molecular and Dissociated Water, M. Akbulut, Rutgers University
31. Water Vapor Uptake of a Stainless Steel Cell at 150 ºC, R. Benson, Johns Hopkins University Applied Physics Laboratory
32. Effects and Optical Properties of Thin Water Films Condensed at Cryogenic Temperatures, B. Wood, Calspan/AEDC
33. A Review of Techniques for the Controlled Delivery of Water Vapor to Vacuum Systems, L. Hinkle, MKS Instruments
34. Water Speed Testing of High Vacuum Pumps and Water Traps, S. Matte, CTI Cryogenics
35. The Influence of Water on the Ultimate Pressure and the Adsorption Capacity of Low Temperature Surfaces, M. Rao, CEBAF
36. Interactions of Trace Moisture with the Components of a Gas Distribution System, J. McAndrew, Air Liquide
